# When It Doesn't Fit: Congenital Anomalies of the Choledochus

**DOI:** 10.1055/s-0039-1693998

**Published:** 2020-11-09

**Authors:** Helena Reusens, Mark Davenport

**Affiliations:** 1 Department of Paediatric Surgery, King's College Hospital, London, United Kingdom

**Keywords:** choledochal cyst, congenital choledochal malformation, hepaticojejunostomy, epidermoid cyst

## Abstract

**Introduction** Congenital choledochal malformations (CCMs) are characterized by intra- and/or extrahepatic bile duct dilatation. Five basic types (1–5) are recognized in Todani's classification and its modifications, of which types 1 and 4 typically have an associated anomalous pancreatobiliary junction and common channel (CC). We describe two cases with previously undescribed features.

**Case Report** 1 Antenatal detection of a cyst at porta hepatis was made in an otherwise normal girl of Iranian parentage. She was confirmed to be a CCM (20 mm diameter), postnatally, with no evidence of obstruction. Surgical exploration was performed at 12 weeks. She had an isolated cystic dilatation of the right-hepatic duct only. The left-hepatic duct and common bile duct (CBD) were normal without a CC. Histology of the resected specimen showed stratified squamous epithelium.

**Case Report 2** A preterm (31 weeks of gestation) boy of Nigerian parentage was presented. His mother was HIV + ve and he was treated with nucleoside reverse transcriptase inhibitors following birth. He had persistent cholestatic jaundice and a dilated (10 mm) bile duct from birth. Although the jaundice resolved, the dilatation persisted and increased, coming to surgery aged 2.5 years. This showed cystic dilatation confined to the common hepatic duct, and otherwise normal distal common bile duct and no CC.

**Result** Both underwent resection with the Roux-en-Y hepaticojejunostomy reconstruction to the transected right-hepatic duct alone in case 1, leaving the preserved left duct and CBD in continuity, and to the transected common hepatic duct in case 2.

**Conclusions** Neither choledochal anomaly fitted into the usual choledochal classification and case 1 appears unique in the literature.

## Introduction

Congenital choledochal malformation (CCM) simply implies dilatation of the extra- and/or intrahepatic biliary tract in the absence of obstruction. The actual pathogenesis is not known for certainty, although most seem to be related to a degree of distal stenosis leading to proximal dilatation. 1

We have used our own Kings' College Hospital classification, which is a simplification of Todani's classification, a modification of previous classifications. The commonest are characterized as a cystic extrahepatic dilatation (type 1c), a fusiform extrahepatic dilatation (type 1f), and the combination of either with intrahepatic duct dilatation (type 4). In most of them, there is also an accompanying pancreaticobiliary junction malformation and long common channel (CC) which has the potential for free intermixing of bile and pancreatic juice.

We describe two patients with atypical morphology that did not conform to any of the usual descriptions.

## Case Reports

**Case 1** : the first case concerns a female infant of Iranian parentage born at term. Diagnosis of a cyst at the porta hepatis was made on an antenatal scan just before birth after arrival in the United Kingdom. Previous scans from abroad were not available. She developed jaundice with a maximum total bilirubin of 380 µmol/L that settled spontaneously. Alpha feto-protein (AFP) level was 756 kIU/L. Ultrasound and subsequent magnetic resonance cholangiopancreatography (MRCP) showed a cyst with dimensions 20 mm × 16 mm at the porta hepatis, without evidence of proximal intrahepatic biliary dilatation.

**Fig. 1:**
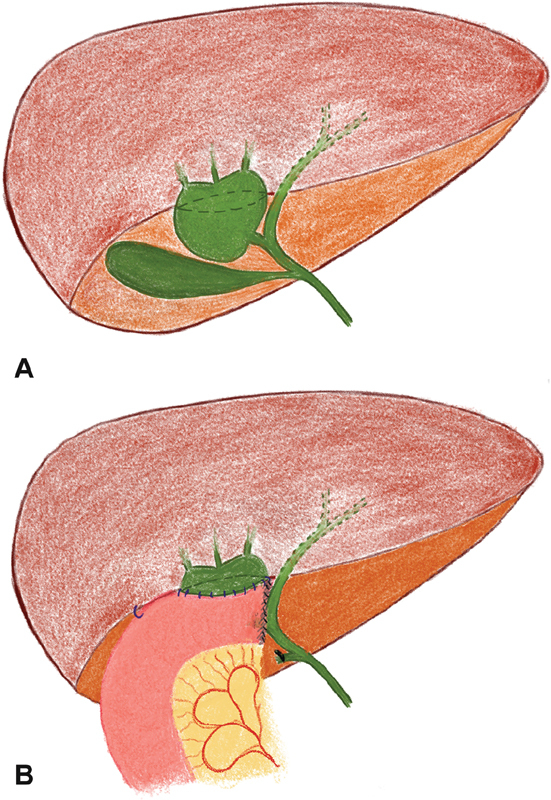
Case 1: ( **A** ) schematic drawing of the choledochal malformation of the right-hepatic duct; ( **B** ) postoperative right-hepatic duct jejunostomy.

Surgical exploration and cholangiography was performed at 12 weeks and showed cystic dilatation confined to the right-hepatic duct with an entirely normal left-hepatic duct draped around and with a low insertion of a cystic duct into a normal-looking common bile duct (CBD; Fig. 1A ). Intraductal pressure was measured at 5 mm Hg (normal). There was no evidence of a CC on the cholangiogram. Bile amylase was 4 IU/L and CA19-9 was 89,400 kU/L.

Following cholecystectomy, the cyst was detached from the CBD and the transected proximal part with its draining segmental ducts anastomosed to a Roux's loop ( Fig. 1B ). The left duct and CBD were left intact. Histopathology showed a cyst lined by nonkeratinising stratified squamous epithelium. Follow-up up to 1.5 years had been unremarkable.

**Fig. 2:**
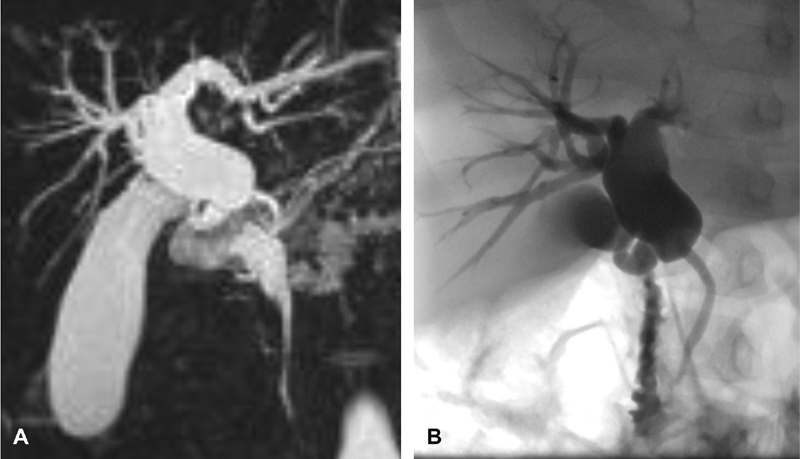
Case 2: preoperative MRCP ( **A** ) and ERCP ( **B** ) images of dilated proximal CBD. Note the normal distal CBD and absence of a CC. CBD, common bile duct; CC, common channel; MRCP, magnetic resonance cholangiopancreatography.

**Case 2** : The second case was a boy of Nigerian origin, born prematurely at 31 weeks, who was presented with transient conjugated jaundice and was found to have a dilatation (10 mm) of the proximal CBD. His mother was HIV + ve (low-risk retrovirus positive), and he was treated with a nucleoside reverse transcriptase inhibitor since birth. At birth, he had positive anti-HIV antibodies but DNA was not detectable. Images from the preoperative MRCP and ERCP are shown in Fig. 2 .

Jaundice resolved but the dilatation persisted and increased over time, so he underwent surgical exploration at the age of 2.6 years. This showed a cystic dilatation extending proximally from the junction of cystic duct to the common hepatic bile duct (CHD). Intracystic pressure was measured at 20 mm Hg (normal < 5 mm Hg). The remaining distal CBD appeared normal and there was no evidence of a CC. Bile amylase was 5 IU/L and CA 19.9 was 9,600 kU/L. Both cyst and gallbladder were resected and a hepaticojejunostomy en-Roux performed. Histopathology showed a dilated bile duct lined by columnar type biliary epithelium with mural fibrosis but with no significant inflammation.

He has had 2 years of follow-up with normal serial ultrasound scans.

## Discussion

CCMs are rare with most infants and children (> 80%) presenting as types 1c, 1f, or 4. Most cases of extrahepatic dilatation are stereotypical with two basic morphologies. The classical choledochal cyst (type 1c) involves the entire CBD, and CHD ending abruptly in what can be a filamentous connection with the pancreatic duct. Alternatively, the dilatation appears fusiform, of smaller diameter, with a more gradual termination and junction with the pancreatic duct.

Unilateral cystic dilatation of the hepatic duct is exceptional with perhaps only two reported cases in the literature. Prekop et al reported a 14-month-old boy with a large “cystic malformation of the right-hepatic duct,” with little more clinical detail including nature of epithelial lining. 2 Gidi et al reported a 56-year-old woman with cystic dilatation of the right-hepatic duct who underwent a resection of the cyst with right hepatectomy to achieve complete resection. Histopathology was similar to our own, although it was described as extensive squamous metaplasia with areas of columnar epithelium rather than de novo stratified squamous epithelium characteristic of ours. 3 As normal bile duct consists of vascularized, innervated fibrous tissue, lined by a single layer of tall columnar epithelium, we feel that ours is likely to be congenital rather than acquired metaplasia. 1

The lining and situation in the porta hepatis in our first case do appear similar to that of the squamous-lined cystic dilatation described by Chiu et al in a 6-month-old boy. 4 This malformation was antenatally detected and, at operation, found to arise from the common hepatic duct with separate entry of both right- and left-hepatic ducts. Finally, Kwon et al described a diverticulum of the bile duct (type 2 CCM) with stratified squamous epithelial lining in a 63-year-old man. 5 Metaplastic squamous lining often associated with recurrent infection and stones can lead to malignant change. 6 Certainly, the age of our child implies a congenital rather than metaplastic origin for the squamous nature of the epithelium.

The second case, while nominally a type 1c, is distinctly unusual with sparing of the distal duct and no CC. The high-intracystic pressure of 20 mm Hg suggests an obstructive etiology, and there was no suggestion of extrinsic compression as might be found with a healed spontaneous perforation. 7 It is interesting to note that this child was delivered from an HIV + ve mother, though it is difficult to find real evidence of cause and effect. 8

We have previously reported a series of CCM where we measured CA 19.9 in bile to try and identify a cohort who might have particular predisposition to dysplasia and perhaps later malignancy. 9 We found that it was invariably raised and often in very-high concentration but bore no relationship with simultaneous obtained bile amylase and choledochal pressure. Indeed, it could be localized on staining to otherwise normal biliary epithelium. CA 19.9 levels in both cases were raised, although whether that represents normal biliary tract epithelium elsewhere or from the cyst is not known.

Surgical reconstruction was bespoke in case 1 with preservation of the left-sided biliary drainage but more typical in case 2. Primary duct to duct anastomosis seemed possible in that case but was rejected, as it was felt to be less safe than a standard Roux's loop.

## Conclusion

In conclusion, neither case fits comfortably into the standard CCM classifications and both present different perspectives on possible etiological factors. 10

## Notes

New Insights and the Importance for the Pediatric Surgeon We describe two variants of choledochal malformations that do not fit the standard classifications. One case appears unique consisting of a unilateral cystic dilatation of the right-hepatic duct, surgically resected and anastomosed to a jejunal loop with preservation of the left-sided biliary drainage. The cyst was lined with nonkeratinising stratified squamous epithelium which is extremely rare in choledochal malformations.
